# The Sooner, the Worse? Association between Earlier Age of Sexual Initiation and Worse Adolescent Health and Well-being Outcomes

**DOI:** 10.3389/fpsyg.2017.01298

**Published:** 2017-07-27

**Authors:** Alfonso Osorio, Cristina Lopez-del Burgo, Silvia Carlos, Jokin de Irala

**Affiliations:** ^1^Institute for Culture and Society, University of Navarra Pamplona, Spain; ^2^Instituto de Investigación Sanitaria de Navarra, Navarra Institute for Health Research Pamplona, Spain; ^3^School of Education and Psychology, University of Navarra Pamplona, Spain; ^4^Department of Preventive Medicine and Public Health, University of Navarra Pamplona, Spain

**Keywords:** adolescents, sexual initiation, sexual health, adolescent well-being, El Salvador, Peru

## Abstract

This cross-sectional study assesses the association between age of sexual initiation during adolescence and a selection of well-being outcomes regarding that first relationship. High-school adolescents from El Salvador (2,686) and from Peru (3,399) replied to a paper-pencil questionnaire. Those who were sexually initiated replied to several questions regarding their age at sexual initiation, condom use, satisfaction and reasons/circumstances for that sexual relationship. Approximately 19% of participants were sexually initiated (*n* = 1,179). After retaining participants with valid responses and with sexual initiation ages between 13 and 17, the final sample for this paper consisted of 996 sexually initiated participants (526 Salvadorians and 470 Peruvians). Multiple logistic regression analyses showed that those who initiated sex at earlier ages had worse outcomes compared to those who initiated at older ages. Specifically, they had lower odds of having used a condom, of having good memories of that experience and of having had that first relationship because they were in love. Conversely, they had higher odds of having had that first sexual relationship as a result of peer pressure (“Most of my friends already had sex”), because of partner pressure (“I was afraid to lose him/her,” “My partner told me he/she would leave me” or “I did not know how to say no to a person who insisted”), or as a consequence of different forms of impaired autonomy (“I was under the influence of alcohol or drugs” or “As a consequence of seeing sexual images”). Results show that sex at earlier ages is associated with worse adolescent health and well-being outcomes.

## Introduction

Minors who start having sex at earlier ages, compared to those who start later, have higher odds of several harmful outcomes such as engaging in unprotected sex (Kirby, 2002; Ma et al., 2009; Finer and Philbin, 2013; Crosby et al., 2015; Shrestha et al., 2016), having an increased number of sexual partners or of casual sexual partners (O’Donnell et al., 2001; Sandfort et al., 2008; Ma et al., 2009; Crosby et al., 2015; Heywood et al., 2015; Shrestha et al., 2016), perpetrating or being a victim of forced sex (O’Donnell et al., 2001; Shrestha et al., 2016), engaging in paid sex (Shrestha et al., 2016), having sex while drunk or drugged (O’Donnell et al., 2001; Sandfort et al., 2008), pregnancy (O’Donnell et al., 2001; Kirby, 2002; Ma et al., 2009; Crosby et al., 2015; Shrestha et al., 2016), abortion (Ma et al., 2009), STIs (Ma et al., 2009; Wand and Ramjee, 2012; Crosby et al., 2015; Lee et al., 2015; Shrestha et al., 2016), depression (Spriggs and Halpern, 2008), behavior problems (Udell et al., 2010), not attending tertiary education (Parkes et al., 2010) or regretting the age of sexual initiation (Cotton et al., 2004). However, some studies describe adolescent sexual initiation as a rite toward maturity or a transitional step taken by those who are ready (Ott et al., 2006; Masters et al., 2008).

Due to these different views on adolescent sexual behavior a debate has been established regarding which messages should be conveyed to youth. Some prioritize the invitation to children and adolescents to delay their sexual initiation in order to avoid risks (Halperin et al., 2004, 2011). Others maintain that this is unrealistic, and suggest to focus on risk reduction strategies such as condom use or partner reduction (Jemmott et al., 1998; Underhill et al., 2007; Jemmott et al., 2010). And there are those that simply explain both strategies without specifically focusing on any (Centers for Disease Control and Prevention, 2016).

Some of the risks associated with early sexual debut can be partly mediated by other variables such as number of lifetime sexual partners or condom use (DiClemente et al., 2005; de Sanjose et al., 2008; Wand and Ramjee, 2012; Oliveira-Campos et al., 2013). However, other risks might be intrinsic to the fact of initiating sex at a younger age (Stöckl et al., 2013). The lack of maturity could increase the odds of making less autonomous and sound decisions, for example related to sexuality (Patton and Viner, 2007; Casey et al., 2008).

Moreover, it seems that adolescents (or at least adolescent women) are biologically more susceptible to STIs. It has been recently explained that adolescent girls may have a higher risk of HIV infection as a result of their immature cervix. The cervix has a greater amount of genital mucosa with a high susceptibility to the virus (Dellar et al., 2015). Young people are also less likely to be at antiretroviral treatment and therefore, virally suppressed, which means they would have a higher transmission risk (World Health Organization, 2010, p. 57; Wringe et al., 2012).

In order to add information to this debate, we studied the association between age of sexual initiation during adolescence and a selection of well-being outcomes regarding that first relationship. One outcome was more related to sexual health (condom use) and the remaining ones were more related to feelings (5 variables measuring satisfaction) and circumstances related to sexual initiation (12 reasons for having sex). Specifically, we worked with the following research questions:

(1) Is there an association between age of sexual initiation during adolescence and *condom* use at the first sexual relationship?
(2) Is there an association between age of sexual initiation during adolescence and *satisfaction* with the first sexual relationship?
(3) Is there an association between age of sexual initiation during adolescence and the *reasons* involved in the first sexual relationship?

## Materials and Methods

The study is based on Project YOURLIFE, an international cross-sectional and longitudinal study on youth, love and sexuality. This project has been described in more detail elsewhere (de Irala et al., 2009; Osorio et al., 2012, 2015; Carlos et al., 2016).

### Sample

Project YOURLIFE is being conducted in different countries worldwide. In its first phase, it was implemented in the Philippines, El Salvador, Peru and Spain. For the purposes of this study, the cross-sectional data from El Salvador and Peru were used. Within each of these two countries, a multi-stage sampling of clusters of public and private schools was performed.

In El Salvador, we randomly selected 30 public and private high schools from San Salvador, Santa Ana and San Miguel (the three main urban areas of the country). In Peru, we randomly selected 62 public and private high schools from the whole country. Within each school, we asked all 13- to 18-year-old students to participate. We expected to recruit around 3,000 participants in each country.

These sample sizes were chosen taking into account approximate sample size estimation criteria (Hosmer and Lemeshow, 1989; Vittinghoff and McCulloch, 2007). We based our estimates on the criteria that 10 respondents with the least frequent outcome would be needed for each parameter included in a statistical model used to adjust for confounding. With these sample sizes we expected to obtain sufficient statistical power to account for a considerable amount of variables in a given model.

For this paper, we only chose sexually initiated respondents who had their first sexual relationship between 13- and 17-years-old. We excluded those who initiated sex before 13 or at 18 because the frequencies were too low to have sufficient statistical power. We also excluded respondents who did not report the age of sexual initiation (this was the case in about 10% of the sexually initiated participants).

### Questionnaire and Variables

The questionnaires, written in Spanish, had close-ended questions. A pilot study was previously conducted, and adjustments were performed in order to improve comprehension and to fit in a 45 min classroom session.

The questionnaire requested information on whether the participants had ever had sexual relationships, defined as ≪complete sexual relationships, also known as “making love,” “having sex”≫. Adolescents who had never had sexual relationships were excluded from the analyses of this paper. Those who had ever had sex were asked several questions regarding their first sexual relationship: age at that relationship, condom use, satisfaction and reasons for having had that relationship. These questions are detailed in **Table 2**, and they were to be replied in a yes/no format (except for the age of first sex, which was answered in a free format).

Socio-demographic questions were also asked, as well as other questions (not relevant for this paper) about sexuality (opinions, attitudes, and behavior), free time activities, and communication with parents.

### Data Collection

The study was run using standardized data-collection protocols (de Irala et al., 2009; Osorio et al., 2012, 2015; Carlos et al., 2016).

Within each country, local collaborators went to the schools to apply the questionnaire. To promote the adolescents’ sense of privacy and their willingness to disclose sensitive information (Tourangeau and Yan, 2007), questionnaires were implemented in the schools (that is, away from parents), and by unknown persons (not the participants’ teachers).

Schools managed parental consent according to their local laws and policies (Ruiz-Canela et al., 2013). Voluntary and anonymous participation were guaranteed. Students were also explained that they could leave the room, leave the questionnaire unanswered, and/or leave any question unanswered if they wished. They were informed as well that replying to the questionnaire was considered as consenting to participate in the study.

The study was approved by the Ethics Committee of the University of Málaga (Spain). It also obtained permission from the Ministry of Education of both countries (El Salvador and Peru).

### Analyses

Bivariate linear trends were measured through simple linear regressions.

Multivariate non-conditional logistic regression models were fit with age of sexual initiation as the main independent variable and different outcomes as dependent variables. In the models we also introduced the main socio-demographic variables as possible confounders: sex, current age (in models regarding satisfaction of first sex only), country, economic status, religiosity, and school type (public/private).

In each model, those who responded “I don’t know” and those who did not respond were considered as missing and therefore not included in the analyses. However, we also repeated all the analyses with different approaches for missing values. In the first approach for analyzing missing values, “I don’t know” responses were included in the least frequent category. For example, those who said they used a condom were compared to those who replied they didn’t use it and those who replied “I don’t know.” In the second approach, all the “I don’t know” responses and non-responses were included in the least frequent category. For example, those who said they used a condom were compared to all the other responses (those who said they did not use it, those who replied “I don’t know,” and those who did not respond).

In addition, all analyses were repeated separately for each country and for each sex.

All analyses were performed using Stata 12; *p* < 0.05 was considered statistically significant.

## Results

The questionnaire was responded by 6,085 adolescents aged 13–18 (2,686 from El Salvador and 3,399 from Peru). Approximately 19% (*n* = 1,179; 591 Salvadorians and 588 Peruvians) had ever had sexual relationships. After dropping those who didn’t report their age at sexual initiation and those who reported an age below 13 or an age of 18, we obtained a final sample of 996 adolescents (526 Salvadorians and 470 Peruvians) for this paper.

**Table 1** shows the main socio-demographic data of these adolescents. Most sexually experienced participants were males (68.4%), and they had a mean age of 15.8 years. They were mostly Catholic, studied in public schools and lived in families with a middle socioeconomic level. We do not report the mean age of sexual initiation because the use of the mean age has been found to be misleading (de Irala et al., 2011, 2014). Conversely, the table contains the percent of participants who initiated at each age.

**Table 1:** Socio-demographic characteristics of the participants.

Features	El Salvador %	Peru %	Total %
	(*N* = 526)	(*N* = 470)	(*N* = 996)
**Sex**			
Women	28.1	35.5	31.6
**Age (years)**			
13	5.3	0.4	3.0
14	14.5	11.7	13.2
15	19.2	30.6	24.6
16	20.7	38.5	29.1
17	23.4	16.0	19.9
18	16.9	2.8	10.2
Total	100.0	100.0	100.0
**Religion**			
No religion	18.4	13.8	16.2
Catholic	47.9	69.1	58.1
Protestant	28.7	12.3	20.8
Other^a^	5.1	4.8	4.9
Total	100.0	100.0	100.0
**Type of school**			
Public	58.0	50.9	54.6
**Socioeconomic level**			
Low	17.3	12.9	15.2
Middle	63.1	67.1	65.0
High	19.6	20.0	19.8
Total	100.0	100.0	100.0
**Age of sexual initiation (years)**			
13	27.2	14.7	21.3
14	23.0	28.1	25.4
15	22.8	35.7	28.9
16	16.9	17.9	17.4
17	10.1	3.6	7.0
Total	100.0	100.0	100.0


^a^*Other religions include Jehovah’s Witnesses, Mormons, Jews, Muslims, Orthodox and “other.”*

**FIGURE 1:**
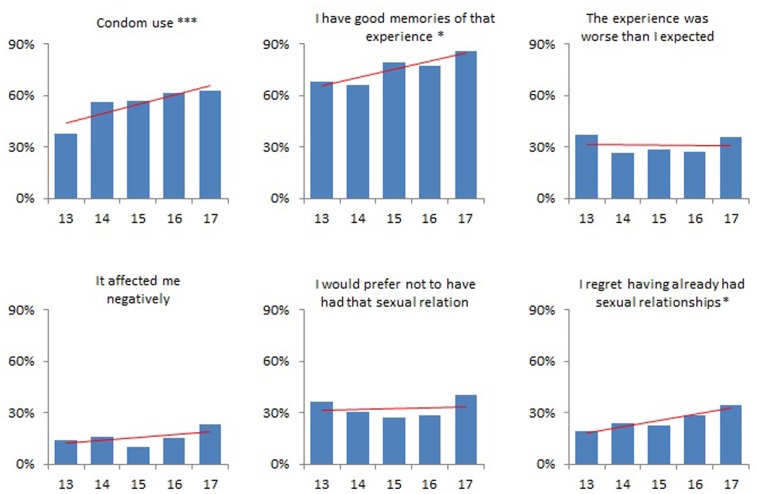
Condom use at sexual initiation and satisfaction on first sex, by age of sexual initiation. *P*-value for linear trend: ^∗^*p* < 0.05; ^∗∗∗^*p* < 0.001. Data for adolescents initiating sex at age 17 are unstable due to a smaller sample size.

**FIGURE 2:**
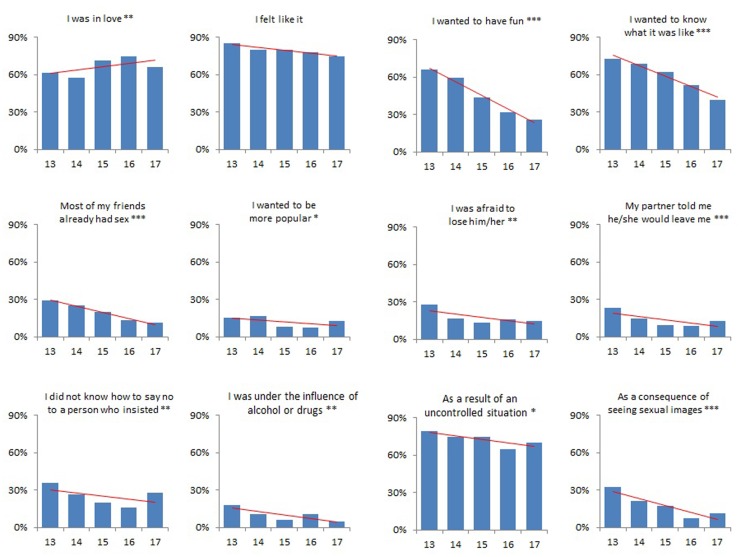
Reasons for first sex, by age of sexual initiation. *P*-value for linear trend: ^∗^*p* < 0.05; ^∗∗^*p* < 0.01; ^∗∗∗^*p* < 0.001. Data for adolescents initiating sex at age 17 are unstable due to a smaller sample size.

Some well-being outcomes varied across the different ages of sexual initiation. **Figure 1** shows the trends in condom use at first sex as well as in feelings related to the satisfaction concerning that first sexual experience. Participants initiating sex at 17-years-old, compared to those initiating at 13, reported a significantly higher frequency of: condom use at first (62% vs. 38%), good memories of their first sex (86% vs. 68%) and regret over having already had sex (34% vs. 19%). **Figure 2** shows the trends over different ages of several reasons for having had their first sex. Love was reported as a reason among participants initiating at 17 significantly more often than among those initiating at 13 (66% vs. 62%). On the contrary, other reasons were more frequent among those initiating at earlier ages.

Multiple logistic regressions (**Table 2**) show that some of these trends of condom use, satisfaction and circumstances concerning first sex are statistically significant even after adjusting for several confounders. Specifically, these regressions show that initiating sex at an older age was associated with more condom use, better memories of that experience, a higher frequency of referring love as a reason for having had first sex, and a lower frequency of several less positive reasons for having had that first sex, such as having first sex for fun, as a result of curiosity, because most of friends had had sex, because of being afraid of losing the partner, because the partner had said they would leave the participant, because of partner insistence, because of the influence of alcohol or drugs and as a consequence of seeing sexual images.

**Table 2:** Outcomes associated with age of sexual initiation.

Outcomes^a^	Adjusted OR of each outcome for 1 year increase in age of sexual initiation^b^	*P*-value
**Condom use**	1.33 (1.18–1.50)	<0.001
**Satisfaction with first sex:**		
I have good memories of that experience^c^	1.80 (1.36–2.39)	<0.001
The experience was worse than I expected^c^	0.80 (0.62–1.04)	0.092
It affected me negatively^c^	0.88 (0.65–1.18)	0.390
I would prefer not to have had that sexual relation^c^	0.82 (0.66–1.03)	0.089
I regret having already had sexual relationships	0.97 (0.81–1.16)	0.764
**Reasons for first sexual relationship**		
I was in love	1.14 (1.00–1.31)	0.048
I felt like it	1.00 (0.85–1.17)	0.989
I wanted to have fun	0.70 (0.61–0.81)	<0.001
I wanted to know what it was like	0.78 (0.69–0.90)	<0.001
Most of my friends already had sex	0.75 (0.62–0.90)	0.002
I wanted to be more popular	0.87 (0.70–1.07)	0.184
I was afraid to lose him/her	0.76 (0.64–0.92)	0.004
My partner told me he/she would leave me	0.72 (0.59–0.88)	0.002
I did not know how to say no to a person who insisted	0.79 (0.67–0.92)	0.003
I was under the influence of alcohol or drugs	0.72 (0.57–0.90)	0.004
As a result of an “uncontrolled situation”	0.89 (0.78–1.03)	0.109
As a consequence of seeing sexual images	0.70 (0.58–0.84)	<0.001


^a^*Each row is a logistic regression where the variable in the first column is the dependent variable. The independent variable is always the age of sexual initiation (as a continuous variable). ^b^Adjusted odds ratios and their respective 95% Confidence Intervals. Analyses are adjusted by sex, country, religiosity, type of school and socioeconomic level. Models regarding satisfaction of first sex are additionally adjusted for age when filling the questionnaire. ^c^This variable is available for Peruvian participants only*.

Separated analyses for each country and for each sex were performed. Results were similar, though we found fewer statistically significant results because of the smaller sample sizes. Sensitivity analyses with different approaches to analyze missing values (see Materials and Methods) showed similar results too (data not shown).

## Discussion

These results are a contribution to the body of knowledge suggesting that adolescents who have their first sexual relationship at earlier ages are more frequently subject to a selection of negative well-being outcomes.

Regarding condom use, the results from El Salvador and Peru are similar to those found in previous studies from other countries: condoms are more frequently used when sexual initiation occurs at an older age (Ma et al., 2009; Finer and Philbin, 2013; Crosby et al., 2015; Shrestha et al., 2016). This is not surprising, given the fact that younger adolescents are less mature and more impulsive. Actually, it is known that, during adolescence, there is a gap between the relatively slower maturation of the prefrontal cortex, responsible for planning and logical decision making, and the limbic system were emotions and rewards are generated and sought (Patton and Viner, 2007). In fact, in previous publications from this same study in El Salvador (de Irala et al., 2008, p. 59), Peru (Corcuera et al., 2010, p. 111) and Spain (Calatrava, 2010 Unpublished, p. 77), we had shown that the difficulty to obtain condoms or the lack of knowledge were not the most important reasons stated by adolescents when condoms were not used in their first sexual relationships. Conversely they overwhelmingly responded that non-condom use was due to the fact that the sexual encounter was not planned or expected. Similar results were found in another study among Irish youth (Layte et al., 2006, p. 28).

Within the issue of satisfaction with the first sexual encounter, the results are also consistent with previous research. A study in South Africa found that early sexual initiation was associated not only with less condom use and more sexual partners, but also with not feeling that they had been ready and wanted to have sex at the time of sexual initiation (Harrison et al., 2005). In our study, adolescents who initiated sexual relationships at an older age had better memories of their first experience. The remaining 4 associations measuring less satisfaction concerning the first sexual experience were not statistically significant although point estimates were in the expected direction yielding better estimates at older ages.

Our study also finds an association between age of sexual initiation and the circumstances and reasons for having had first sex. Those who initiated at older ages reported love more frequently and reasons related to lack of personal autonomy less frequently: peer pressure, partner pressure, substance use and sex because of having seen sexual images. This is consistent with the British results from the National Survey of Sexual Attitudes and Lifestyles (NATSAL). Some studies within this project have found an association between earlier age of first sex and different main reasons for first intercourse having occurred: peer pressure (Wellings et al., 2001) and being drunk (Schubotz et al., 2004). Some of these studies have built a composite measure (“sexual competence”), one of which components is to report autonomous reasons for the first intercourse. Sexual competence was also found to be associated with older age of first sex (Wellings et al., 2001).

The reasons for first sex do have important sexual health implications. For example, the lack of the aforementioned “sexual competence” was associated with a higher risk of having STIs, low sexual function, unplanned pregnancies and non-volitional sex (Palmer et al., 2016). Similarly, intimate reasons for first sex (for example, loving the partner) were associated with using contraceptives and with discussing contraceptive use before first intercourse (Stone and Ingham, 2002). Finally, in the United States, Else-Quest et al. (2005) defined that the first sexual experience occurred in a negative context if (among other reasons) the participant’s main reason for first sex was peer pressure or the influence of drugs or alcohol. Such negative context was associated with sex dysfunctions, sex guilt, STIs, poorer health, and less life satisfaction.

In summary, our results seem to confirm the association of an earlier age of sexual initiation with a selection of negative outcomes. This seems to pertain to a cross-cultural phenomenon: several important aspects related to sexual initiation in adolescence improve when such initiation takes place at older ages. In this study, what our outcomes have in common is that they are not “long term” negative consequences of sexual initiation rather immediate potential problems resulting from having first sex at that moment.

The main possible limitation of this study is that respondents might have differentially and erroneously recalled events (condom use, satisfaction and reasons for first sex) depending on age of initiation. The worse-case scenario would be that those that had their first sex at older ages might have overestimated their condom use, satisfaction and quality of reasons for first sex compared to those initiating sex at younger ages. We have no reason to believe that this is likely the case in our study. Rather, as the *rosy view* shows, past events tend to be evaluated better (Mitchell et al., 1997). That would imply that those having initiated sex earlier would recall a rosier experience than they really had, and consequently this could imply that their experience might have been even worse than what their replies show.

As in any cross-sectional study, it is important to correctly interpret the associations because the time sequence between independent and dependent variables has to be well established and/or understood. The interpretation of the association between earlier sexual initiation and less condom use or less satisfaction is straightforward. These outcomes are the result of younger age at sexual initiation. The association between earlier sexual debut and variables such as peer or partner pressure or substance use (reasons for first sex), is more correctly interpretable by affirming that they suggest that earlier sexual initiation takes place more frequently under those cited circumstances.

As for the strengths, the study consists of two large representative samples of school-attending adolescents in El Salvador and Peru. The results have been adjusted for possible confounders and are consistent across countries, and sensitivity analyses show similar results.

Our results seem to confirm that earlier sexual initiation is associated with worse outcomes related to the sexual health and well-being of adolescents. There is room and epidemiological reasons to empower youth (through family, schools, social media and other agents) to delay their sexual debut so as to be better prepared to be more in charge of their reproductive health when sexually active at later ages, as well as to make better decisions concerning the consequences of having sex.

## Ethics Statement

The study was approved by the Ethics Committee of the University of Málaga (Spain). Survey procedures were designed to protect student privacy by ensuring voluntary and anonymous participation. Students were informed that their participation was voluntary and anonymous, and that they could leave the room and/or leave the questionnaire unanswered. They were also told that they could leave any question unanswered if they wished. They were also informed that answering the questionnaire was considered as consent to participate in the study.

## Author Contributions

All authors participated in the design and implementation of the project. AO and JdI designed this specific study. AO performed the analyses and wrote the initial draft. All authors revised the draft, made substantial contributions and approved the final manuscript.

## Conflict of Interest Statement

The authors declare that the research was conducted in the absence of any commercial or financial relationships that could be construed as a potential conflict of interest. The reviewer KR and handling Editor declared their shared affiliation, and the handling Editor states that the process nevertheless met the standards of a fair and objective review.
